# Preventing sexual violence in Vietnam: qualitative findings from high school, university, and civil society key informants across regions

**DOI:** 10.1186/s12889-023-15973-5

**Published:** 2023-06-10

**Authors:** Kathryn M. Yount, Katherine M. Anderson, Quach Thu Trang, Irina Bergenfeld

**Affiliations:** 1 Hubert Department of Global Health, Rollins School of Public Health, Emory University, 1518 Clifton Rd NE, Atlanta, GA 30322 USA; 2 Department of Sociology, Emory University, Atlanta, GA USA; 3 Department of Behavioral, Social, and Health Education Sciences, Rollins School of Public Health, Emory University, Atlanta, GA USA; 4 Center for Creative Initiatives in Health and Population, Hanoi, Vietnam

**Keywords:** Adolescents, Civil society organizations, Implementation science, Primary prevention, High school, Sexual violence, Southeast Asia, University, Vietnam, Youth

## Abstract

**Background:**

Sexual violence by young men against women is common, but efficacious primary prevention interventions tailored to men are limited in low- and middle-income settings like Vietnam. GlobalConsent, a web-based sexual violence prevention intervention tailored to university men in Hanoi, is efficacious. Implementation research is needed to understand facilitators and barriers to scaling GlobalConsent and prevention programs generally. We conducted qualitative research with key informants from three youth-focused organizational settings to understand the context of implementation in Vietnam.

**Methods:**

Interviews with university (*n* = 15), high-school (*n *= 15) and non-governmental (*n* = 15) key informants focused on perceptions about sexual violence among young people and prevention programming. Four focus group discussions with 22 interviewed informants, following the Consolidated Framework for Implementation Research, asked about facilitators and barriers to implementing GlobalConsent. Narratives were transcribed, translated, and coded inductively and deductively to identify salient themes.

**Results:**

Outer-setting influences included greater expectations for sex among young people alongside norms favoring men’s sexual privilege, ostensibly ambiguous and lax laws on sexual violence, government ministries as bureaucratic but potential allies, external subject-matter experts, and the media. Inner-setting influences included variable cultures regarding openness to discuss sexual violence and equitable gender norms, variable departmental coordination, limited funding and ‘red tape’ especially in public institutions, inconsistent student access to technologies, and limited time and competing priorities among students and teachers. Several actors were considered influential, including institutional leaders, human-resource staff, the Youth Union, and student-facing staff. Important characteristics of individuals for implementation included subject-matter expertise, science or social science training, younger age, engagement in social justice related activities, and more open attitudes about sex. Regarding characteristics of sexual violence prevention programming, some participants preferred online formats for busy students while others suggested hybrid or in-person formats, peer education, and incentives. Participants generally accepted the content of GlobalConsent and suggested adding more content for women, ancillary support services, and adapted content for high-school students.

**Conclusions:**

Implementation of sexual violence prevention programs in youth-focused organizations in Vietnam requires multilevel strategies that connect outer-setting subject-matter experts with supportive inner-setting leaders and student-facing staff to overcome normative and organizational constraints, and thereby, to deliver institution-wide programming.

**Supplementary Information:**

The online version contains supplementary material available at 10.1186/s12889-023-15973-5.

## Contributions to the literature

- Sexual violence by young men against women is common, but tailored prevention is lacking in LMICs.
- GlobalConsent, a web-based sexual violence prevention program, is efficacious among university men in Hanoi. Understanding facilitators and barriers to scale-up is needed
- Key informants from youth-focused organizations across Vietnam suggest that institution-wide implementation of prevention programs requires multilevel strategies connecting outer-setting experts/allies with inner-setting leaders and student-facing staff to overcome normative and organizational constraints.
- Implementation champions may be younger, urban, subject-matter experts with open attitudes about sex among young people.
- This framework offers novel guidance for implementing sexual violence prevention programs in LMIC settings.

## Introduction

### Sexual violence against young women globally and in Vietnam

Sexual violence includes any sexual act committed against a person without active consent [1, 2]. Sexually violent behavior (SVB) disproportionately occurs by men [3] against women, who account for 89% of sexual-assault victims in some settings [4]. Women’s exposure to SVB often starts at a young age, with 14.9% of women globally experiencing a forced sexual debut [5]. Women victims of SVB have heightened risks of adverse physical, mental, and academic outcomes [6–8], highlighting the need for effective prevention.

In Vietnam, sexual violence against women persists despite legal and policy reform to define it and to support survivors [9]. In 2019, an estimated 9.1% of Vietnamese women 15–19 years old and 18.0% of women 20–24 years old reported experiencing sexual violence since age 15 [10]. A constellation of harmful social norms may contribute to the persistence of SVB in Vietnam. Specifically, changing sexual norms among youth are setting expectations for sex in dating relationships; however, the persistence of inequitable gender norms characterized by masculine privilege, feminine discretion, and victim-blaming normalize men’s sexual coercion and undermine sexual consent [11–13].

### Primary sexual violence prevention interventions with young men

Efficacious interventions to prevent SVB among men remain rare in lower- and middle-income countries (LMICs) [14, 15]. Evidence from randomized trials of intimate-partner, dating, and sexual violence prevention interventions tailored to men has been mixed and predominantly from U.S. university students [16, 17]. Intervention studies often have suffered from small sample sizes, high attrition, short follow-up periods of six months or less, and heterogeneity in outcome measurement, including an infrequent focus on behavioral outcomes [16].

To address the high prevalence of SVB against young women amidst unfavorable sexual and gender norms in Vietnam, our team adapted [18, 19] an evidence-based intervention developed in the U.S. [20] and tested impacts of the adapted program, GlobalConsent, on SVB and prosocial bystander behavior among university men in Hanoi [9, 21]. Relative to an attention-control condition, initiating GlobalConsent lowered the odds of SVB mainly through increases in knowledge of sexual violence legality and harm and victim empathy, and increased the odds of prosocial bystander behavior directly and indirectly, through increases in knowledge of sexual violence legality and harm and bystander capacities [9, 21]. Research is needed on the implementation and effectiveness of GlobalConsent in universities and its adaptability to other age groups and organizations in Vietnam.

### Implementation of efficacious sexual violence prevention interventions at scale

Systematic implementation is essential to maintain the fidelity and effectiveness of evidence-based interventions (EBIs). Yet, challenges during implementation are common and often attributable to contextual factors [22, 23], resulting in gaps between the impact of the EBI in efficacy trials, effectiveness research, and applied best practice. In response, implementation science involves research to bridge efficacy and effectiveness to improve the public health impact of interventions [24, 25]. The use of implementation science can facilitate the scaling of interventions to organizational, community, and national levels [26–28]. Such scale is vital to initiate population-level change, like that required for the prevention of SVB. However, the field of violence prevention has underemployed implementation science [29], with few published examples of theory-informed intervention implementation [30–35], and fewer specific to sexual violence prevention.

Moreover, numerous theories and frameworks exist within implementation science, but publication on global implementation science is limited [36] and requires integration of global voices and perspectives into existing frameworks [37, 38]. The Consolidated Framework for Implementation Research (CFIR) is a meta-theoretical implementation science framework designed to capture salient factors influencing program implementation (Damschroder et al., 2009). The CFIR includes five domains—the outer setting, inner setting, individual characteristics, intervention characteristics, and implementation process—which themselves include 39 constructs. Researchers select domains and constructs that resonate with a particular research question and targeted implementation outcomes [38, 39]. Since its creation, CFIR has been used in a range of health contexts [39], including internationally and in LMICs [38]. The CFIR recently has been applied to contexts of violence prevention, including IPV screening and prevention [40–42].

### Study aims

The study aims were twofold. First, we explored perceptions among key informants from youth-focused organizations about the nature, scope, and reasons for sexual violence among young people in Vietnam. Second, we explored views among key informants about the facilitators of and barriers to implementing sexual violence prevention programs in these organizational settings. The CFIR guided our investigation to ensure that implementation factors were explored comprehensively. Findings offer insights about the implementation strategies needed to scale-up GlobalConsent and other sexual violence prevention programs in these organizational settings in Vietnam.

## Methods

### Study setting

Vietnam, a lower middle-income country occupying the eastern portion of mainland Southeast Asia, has a population of over 98.5 million [43]. About 85% of the population is of Kinh ethnicity, and 54 ethnic groups are recognized [44]. Although most of the population does not affiliate with a religion, Christians and Buddhists are prominent minority groups [45]. Over the last two decades, Vietnam’s economy has grown 7% annually [46] while poverty has declined [47]. Literacy is almost universal among those 15 years or older [48]; however, gender gaps in schooling persist, with the mean years of schooling still lower for women (8.0) than men (8.7) 25 years or older [49]. That said, gender gaps in schooling attainment are expected to reverse due to faster projected increases in attainment for girls (13.2 years) than boys (12.7 years) of school-entry age in 2021 [49]. Labor force participation rates for adults 15 years or older are high for women (69.6%) and men (79.4%); however, gender gaps in gross national income per capita are large (6,932 for women, 8,826 for men in 2017 Purchasing Power Parity US dollars) [49]. Mobile phones are widespread, with over 141 subscriptions per 100 inhabitants in 2019 [50].

The government of Vietnam has undertaken several legal reforms to prevent sexual violence. Revisions of the Penal Code (2015, 2017) have widened the definition of rape to include “other sexual activities” and have added an article on employment of persons under 16 for pornographic purposes. The Supreme Court issued Resolution 06/2019/NQ-HDTP (2019) to guide interpretation of new terms in the Penal Code, including intercourse with children, by age, within same-sex and opposite-sex relationships, and with a “defenseless” victim. The Ministry of Health issued Decision 3133/2020/QD-BYT (July 2020) to guide health professionals in caring for victims of sexual violence and in examining, documenting, and reporting on cases for investigation processes.

Despite legal reforms, sexual violence victimization persists, and sexual violence prevention programming remains uncommon. Given the availability of efficacious sexual violence prevention programming tailored to young men in Vietnam [9, 21], a useful next step is to understand the factors that key informants identify as influential for the uptake, implementation, and scaling of such programming in these settings.

### Study design

The study design involved semi-structured interviews and focus group discussions with a purposive sample of key informants from universities, high schools, and youth-focused civil society organizations (CSOs) throughout Vietnam. Semi-structured interviews focused on key informants’ perceptions about the context of sexual violence among young people and the prospects for prevention intervention. A subset of interviewed key informants participated in focus group discussions to understand more systematically the facilitators and barriers to implementing GlobalConsent and other sexual violence prevention programming, using the CFIR as a guide [51].

### Study forms

The team developed three study forms—a key informant interview guide, a GlobalConsent viewing guide, and a focus group discussion guide—to facilitate the collection of comparative data from participants across all three institutional settings. Study forms in English are available in the Supplemental Materials.

#### Key informant interview guide

The key informant interview guide asked open-ended questions to elicit perceptions about: gender and sexual norms among youth; the nature and scope of sexual violence among youth; causes and effects of SVB; strategies to prevent SVB; and barriers to and facilitators of efforts to prevent SVB. Questions about programmatic approaches were general, to elicit unframed responses about strategies that were and were not recommended in their regional, organizational setting.

#### GlobalConsent viewing guide

A viewing guide was developed to elicit feedback on GlobalConsent from key informants who agreed to participate in focus groups. The guide provided space to record positive and/or negative impressions of each module, to rate on a scale from 1 to 5 the feasibility of implementing the program at their organization, and the acceptability of the program for different stakeholder groups. Participants were invited to share their personal impressions in more detail in the group discussions.

#### Focus group discussion guide

The focus group discussion guide included questions focused on each of the five CFIR domains of influence in the implementation of EBIs. Focal domains included 1) the outer setting, or the environment external to the implementing organization, including broader policies, norms, and influential organizations; 2) the inner setting, or the organizational environment in which the intervention is implemented, including norms, resources, and priorities; 3) intervention characteristics, including the content, administration, and source; 4) characteristics of individuals, including those of individuals administering and receiving the intervention; and 5) process, including planning and evaluating implementation [52]. For example, questions asked about internal and external barriers and facilitators to the implementation of GlobalConsent at their institutions, program content that might be sensitive or welcomed, and characteristics of GlobalConsent that were perceived to facilitate or to hinder implementation that could be adapted to improve the implementation process.

### Sample eligibility and recruitment

Participants were leaders or other key personnel at universities (administrators, lecturers), high schools (e.g., principals, teachers, parents), or youth-centered CSOs who were engaged in sexual and reproductive health and rights in Vietnam. Individuals in senior positions, with potential influence on programming at their institution, were prioritized for recruitment. An initial list of potential participants was developed from 1) professional networks of the team’s local research collaborators, 2) a Google Scholar search using terms “youth and violence in Vietnam,” and a search of abstracts from the 9^th^ Asian-Pacific Conference on Reproductive and Sexual Health and Rights in Vietnam using the search phrase “youth, students and violence” (APCRSHR 2017). This list then was supplemented using snowball sampling [53] while ensuring variation of participants across sectors and geographical regions. A team member invited potential participants by email until 45 participants were identified across high schools (*n* = 15), universities (*n* = 15), and youth-engaged CSOs (*n* = 15). Women and others from diverse backgrounds were included to ensure representation of those voices. From the 45 interviewed key informants, 32 were invited to participate in focus group discussions (FGDs), of whom 22 agreed to participate. Those who were invited to take part in FGDs had expressed some support for sexual violence prevention programming for young people. Four discussion groups were formed (one with seven high school teachers; two with six and three university lecturers, respectively; one with six CSO staff). Participants were compensated $20 for the key informant interview, $30 for reviewing the GlobalConsent program, and $20 for taking part in a group discussion.

### Data collection

#### General procedures

Invitations to participate in interviews and discussions were sent via email, with the consent form attached and a request to confirm via return email of having read the information and of agreement (with a check box in the consent form) to participate. Interviews and group discussions were held using Zoom audio conferencing software, which ensured a diverse sample across organizations and regions of Vietnam, enabled participants to select a private location from which to participate, and aligned with COVID-19 social distancing guidelines that were in place during the period of data collection. Only facilitators and participants were present at focus groups and interviews. At the time of each interview and group discussion, research staff explained the aims of the study and clarified that private, sensitive information would not be asked. Research staff obtained verbal consent to participate and to be recorded from all participants before all interviews and focus group discussions. Personally identifiable information, including participants’ names and employers, was retained. Several methods were used to protect the privacy and confidentiality of participants, including the use of audio-only interviewing and group discussion, password-protected computer data files, and locked cabinets to store digital recorders (if used). All team members were trained in research ethics.

#### Key informant interviews

Two experienced researchers at the Center for Creative Initiatives in Health and Population (CCIHP) conducted the key informant interviews. Each interview was digitally recorded and lasted 45–90 min. After each interview, the interviewer summarized their observations. All digital recordings were transcribed, and random segments of the transcriptions were verified against the recordings. All transcriptions were translated into English, and random segments of the translations were verified against the transcriptions. Once the transcriptions, translations, and quality checks were complete, the digital recordings were destroyed.

#### Focus group discussions

Consenting participants in the group discussions were asked to view the Global-Consent program and to note their personal perspectives on the viewing guide before attending the group discussion. Before starting each discussion, trained facilitators from CCIHP clarified the guidelines to maintain respect and confidentiality during the discussion. During each discussion, facilitators elicited individual responses to the viewing guide while administering questions from the focus group discussion guide. Discussions lasted 120–150 min. The same process as that used for the key informant interviews was used to transcribe and to translate the digital recordings, with verification of random text segments at each stage to ensure data quality.

### Data analysis

Qualitative data analysis entailed deductive and inductive techniques and a two-step coding process to discern general themes and emergent sub-themes [54]. First, two team members developed a draft codebook based on the interview guides and five CFIR domains. After completing a team-based coding on four transcripts, the codebook was revised iteratively to incorporate emergent themes. Once the codebook was finalized, transcripts were analyzed using MAXQDA qualitative data analysis software [55]. Two doctoral-level analysts with training in qualitative research coded the transcripts using broad, deductive codes first to capture each of the five CFIR domains. The analysts then stratified these coded segments by organizational setting (high school, university, CSO) and applied inductive codes, in which sub-domain themes were identified. The analysts then created narrative summaries and selected exemplar quotations at the deductive CFIR domain level and inductive sub-domain level from participants representing each organizational setting. Triangulation—the comparison of data from multiple sources to enhance the validity of each source [56]—was used to identify overarching and organization-specific themes within each CFIR domain. Triangulation was conducted in a discussion-based manner, first between the two coders, and then within the binational research team.

## Results

### Sample characteristics

Table 1 summarizes the characteristics of the study sample. Overall and across institutional settings, the majority of interview participants were female (77.8%, 73.3%-86.7% across settings), as were the sub-sample of focus group participants (77.3%, 57.1%-88.9%). Among CSO members, the gender distribution of focus group participants was more gender equitable (57.1% female; 42.9% male). Overall, a majority of study participants also were from North Vietnam (53.3% of interview participants, 54.5% of focus group participants). Among university lecturers, a majority of interview participants were from the South (53.3%), and among CSO staff, three each (42.9%) were from the North and Central regions of Vietnam.

**Table 1:** Interview and Focus Group Participants, Vietnam

Interview Participants					
		**High School Teachers** (*N* = 15)	**University Lecturers** (*N* = 15)	**CSO Members** (*N* = 15)	**Total** (*N* = 45)
Gender	Female	11 (73.3%)	11 (73.3%)	13 (86.7%)	35 (77.8%)
	Male	4 (26.7%)	4 (26.7%)	2 (13.3%)	10 (22.2%)
Region	North	9 (60.0%)	5 (33.3%)	10 (66.7%)	24 (53.3%)
	South	2 (13.3%)	8 (53.3%)	3 (20.0%)	13 (28.9%)
	Central	4 (26.7%)	2 (13.3%)	2 (13.3%)	8 (17.8%)
**Focus Group Participants**					
		**High School Teachers** (*N* = 6)	**University Lecturers** (*N* = 9)	**CSO Members** (*N* = 7)	**Total** (*N* = 22)
Gender	Female	5 (83.3%)	8 (88.9%)	4 (57.1%)	17 (77.3%)
	Male	1 (16.7%)	1 (11.1%)	3 (42.9%)	5 (22.7%)
Region	North	4 (66.7%)	5 (55.6%)	3 (42.9%)	12 (54.5%)
	South	2 (33.3%)	3 (33.3%)	1 (14.3%)	6 (27.3%)
	Central	0 (0.0%)	1 (11.1%)	3 (42.9%)	4 (18.2%)

Notes. *CSO* Civil Society Organization

### Overview of emergent themes and their contextualized relationships

**Fig. 1:**
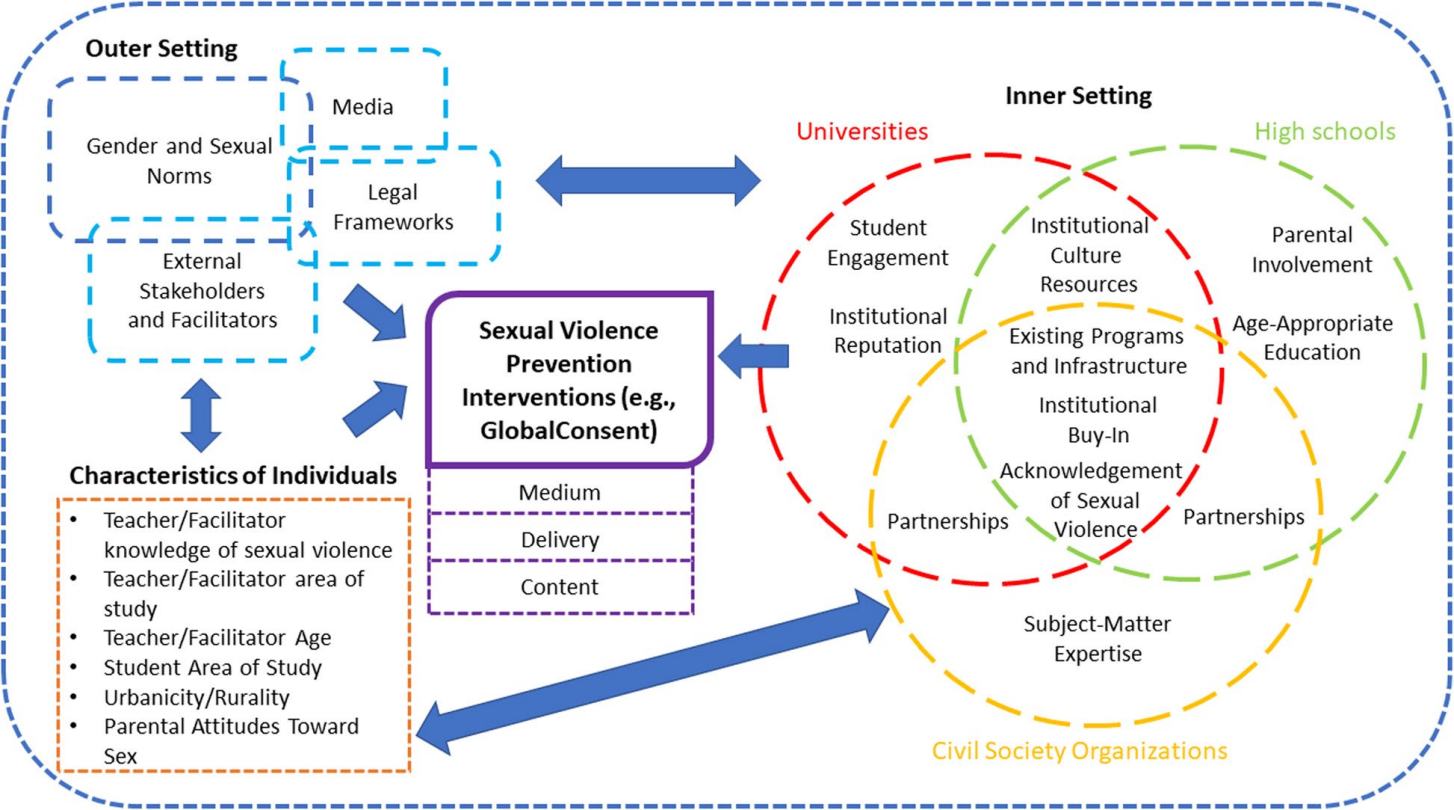
Adapted Consolidated Framework for Implementation Research on Sexual Violence Prevention Interventions in Youth-Focused Organizations in Vietnam

Figure 1 summarizes the contextualized relationships between salient CFIR domains, as articulated by key informants, influencing the implementation of sexual violence prevention programs in high schools, universities, and CSOs in Vietnam. Salient influences emerged in the outer setting, the inner settings of all three organizational environments, the characteristics of individuals within and outside these organizational environments, and the characteristics of sexual violence prevention interventions and their delivery process. Some influences were common to all three organization environments, and some were unique to specific organizational environments. These similarities and distinctions are clarified, below.

### Outer setting influences

#### Changing sexual norms amidst resistant gender norms

Key informants from all organizational settings characterized gender and sexual norms in Vietnam as in a state of transition. While premarital sex among young people was becoming more common and accepted, other entrenched gender norms persisted (Table 2, Quote 2.1). This shift in sexual norms was described as having progressed further in younger than older generations and in more urban than rural areas. Several key informants from the CSO and high school settings, for example, noted parents’ reluctance to talk about sexual matters as a barrier to education and programming (Table 2, Quote 2.2). According to key informants in the CSO setting, parents still monitored and controlled the relationships and sexual activities of daughters more than of sons (Table 2. Quote 2.3). Others, however, felt that even older generations of parents had begun to be more open to discussions of sex and sexuality, particularly as these discussions related to their children’s health.

**Table 2:** Exemplar Quotes: Outer Setting Subthemes

Subtheme	Example
**Changing Gender and Sexual Norms**	2.1 “All parents… want to protect their children, so having premarital sex has always been forbidden. Maybe my way of thinking is a bit old-fashioned like the previous generations, I don't want my children to have experiences that they haven’t fully understood.” -High school Teacher Interview Participant, North Region, Woman
	2.2 “I find that people do not accept their daughters or sons having premarital sex, especially at a young age. They even strongly oppose, force, or prevent it by all means, [even stopping children from being in] love, let alone sex. Some parents even disagree with providing knowledge about sexual and reproductive health, sexuality, and contraception.” -CSO Interview Participant, North Region, Woman
	2.3 “When sexual activity occurs, girls often experience more severe consequences, for example, pregnancy or school drop-out." -CSO Interview Participant, North Region, Woman
	2.4 “Sexual coercion in marriage is sometimes normalized, and women may even think they have to accept to engage in sexual behaviors that they do not want. That is, they do not want to have sex, but still follow the wishes of their husbands, because they are husband and wife.” -High School Teacher Interview Participant, North Region, Woman
**Ambiguity of National Laws on Sexual Violence and Poor Implementation**	2.5 “…the adults (over 18) and the children (under 16) think they can have sex because they are in love. The point is that they do not understand that to protect themselves and others, they must respect the law. Because according to the law, it's illegal to have sex with someone under the age of 16. If you know this law, you will build a good relationship. In many cases, only when wrongdoers went to court did they realize how serious their actions were.” -CSO Interview Participant, Central Region, Man
	2.6 “Sexual violence cases are much more complicated, since the victims need protection. It is also shortcomings in the law, since it missed the part of protection. In addition, there is no clear definition of sexual abuse or sexual assault.” -University lecturer Interview Participant, South Region, Woman
	2.7 “Considering the law of Vietnam, it is almost impossible to take sexual harassment to adjudicate. The maximum penalty for sexual harassment is only two to three years in prison, but to be able to be at that level, it must be very close to sexual assaults… As for the common sexual harassment, even if the case is reported to the authorities, it is almost impossible that the authority will accept the application.” – CSO Interview Participant, Central Region, Woman
**Influences of External Organizations**	2.8 “If we want actually to bring the program into school’s education, the ministry of education and training or the department of education will certainly require expert evaluation first and this will take us a lot of time, steps and procedures.” -High School Teacher Focus Group Participant, Man
	2.9 “Every year, my college cooperates with the Center for Population and Family Planning, and the Center for Social Evil Prevention to organize communication campaigns for students about social evil in general. On the topic of school violence, we invite police officers to come to the college, talk, and share with students. These activities are also within the school's regulations on student affairs.” – University Lecturer Interview Participant, Central Region, Man
**Influences of the Media**	2.10 “…as for information from social networks or the Internet, I think there are quite a lot already. I find that especially in big cities, young people, especially the ones in secondary and high schools, start to have social projects, and they also begin to research this topic and upload posts on social networks.” – CSO Interview Participant, South Region, Woman
	2.11 “Some people go to Internet to find relationships and many times they will become victims of another situation and become the next victims again—it's just that vicious cycle.” -CSO Interview Participant, Central Region, Man
	2.12 “Regarding barriers or obstacles, it's still social media, since it is a double-edged sword. Firstly, not everyone can have access to social media, or to the internet. Not everyone has time to go online to scroll through Facebook to read our latest news and articles. For me, the people who don't have time to do it and don't have the conditions to do it are the people we need to approach most. Because those people are people who don't have much access to the mass media, to both information and knowledge sources on gender and sexual violence. Another difficulty may be the multidimensionality of social media. Every time we upload the information, we also receive many opinions, different kinds of feedback, even some pages, organizations, and activists opposing gender-equality activities also attack us. I think it will create a rather chaotic environment for the audience who are looking for information. The audience who passively receive information can feel confused since they don't know which side to take, they are not sure which side is the right one. Such things disturb information and reduce the effectiveness of our knowledge campaign remarkably." -CSO Interview Participant, Central Region, Woman
	2.13 “Communication has significantly developed over the internet and mutual monitoring. For example, in the past, cases brought to the authorities were not responded to in a timely manner. Now, they bring it to the authorities and they feel unsatisfied, so they put it online, on forums. The community can cause pressure and motivation for authorities to process more effectively. That is also one way that I see it improves and contributes to the effectiveness of child protection work." -CSO Interview Participant, Central Region, Man

Persistent gender norms that bolstered myths about sexual violence, including victim blaming and male sexual privilege within heterosexual relationships, were described as root causes of sexual violence and barriers to addressing it. Several key informants across all organizational settings discussed or gave examples of relationships in which women and girls either did not recognize sexual violence or felt pressured to tolerate sexual violence within the relationship (Table 2, Quote 2.4).

#### Ambiguity of national laws on sexual violence and poor implementation

Closely linked to the influences of gender and sexual norms on sexual violence in adolescent dating relationships, key informants across organizational settings cited the ambiguity of contents and implementation of laws in Vietnam as a challenge to addressing sexual violence. Although the age of consent (16 years) was considered common knowledge, and potential prosecution based on violation of the age of consent was cited frequently (Table 2, Quote 2.5), knowledge was limited about the acts that constituted sexual violence under the law, particularly for those over age 16. Key informants also expressed a lack of clarity about formal avenues of legal recourse, which was perceived to contribute to these challenges. Moreover, some key informants perceived that laws were poorly enforced, further discouraging victims from reporting their experiences to formal authorities (Table 2, Quotes 2.6–2.7).

#### Influences of external organizations

Governmental and non-governmental organizations were highly networked with high schools and universities in the provision and monitoring of education about sexual and reproductive health and sexual violence. The Ministry of Education and Training was viewed as a potential ally in requiring the creation of sexual violence prevention programming, but also was viewed as a source of red tape in the implementation of such programming in public high schools and universities (Table 2, Quote 2.8). When discussed, universities and high schools were partnered with CSOs, medical staff, and the police to provide subject-matter expertise on sexual violence that might be lacking among internal staff at those institutions. Some outside organizations were perceived to be less accessible to adolescents than others due to logistical barriers (Table 2, Quote 2.9).

#### Influences of the media

Finally, traditional and social media arose frequently in discussions of factors influencing the prevalence of and responses to sexual violence. Social media and the internet were viewed as preferred sources of information among adolescents about health and relationships, filling the information gap left by schools, parents, and official government media (Table 2, Quote 2.10). However, technology-facilitated sexual violence was identified almost universally as a growing problem and driver of sexual violence among high school and university students (Table 2, Quote 2.11). Moreover, traditional and social media were believed, on the one hand, to perpetuate harmful norms and rape myths based on the ways in which high-profile cases of sexual violence were covered, but on the other hand, to be potentially important avenues for norms change (Table 2, Quote 2.12–2.13).

### Inner setting influences

Participants shared various thoughts on the implementation inner setting, identified as the internal school or university environment in which GlobalConsent would be administered. Several similarities and differences emerged with respect to the inner settings of high schools and universities.

#### Institutional culture

According to several key informants, the ability of influential actors to advocate for sexual violence prevention depended on the institutional culture around sexual violence and gender. First, several key informants perceived varying institutional openness to address sexual violence among students, staff, and leadership at their institutions. On the one hand, in university settings, concerns about sensitivity and institutional reputation, as well as a belief that sexual violence was uncommon, often were cited as barriers to address the problem (Table 3, Quotes 3.1–3.2). On the other hand, universities in urban settings having social sciences and humanities departments, younger staff, and more international students were characterized as more open to discussions of sexual matters.

**Table 3:** Exemplar Quotes: Inner Setting Subthemes

Subtheme	Example
**Institutional Culture**	3.1 “Frankly speaking, I think the HR officer had the idea that there is no such thing as sexual violence in schools, and if it happens outside the campus and it has nothing to do with the university, then the university should stay out of it because it’s way too sensitive to deal with. That’s why they think the term “sexual violence” is too much. They think that there’s no sexually violent acts committed in the campus, and harassment is just like boys teasing girls, or texting with sexually suggestive messages. They know it’s a thing in students, but to them, physically harmful assault doesn’t happen within the so-called campus, and even if it happens outside the campus, they’re not equipped enough to deal with such case.” -University Lecturer Interview Participant, South Region, Man
	3.2 “According to the sharing of the deputy director of [local university], there is one thing that I observe. Nowadays, universities are very interested in their images. It is not just about a clean environment, or many successful students but a healthy learning environment in terms of caring for both the mental and physical health of their students.” -CSO Interview Participants, North Region, Woman
	3.3 “In terms of culture, there is no mention of what men need to do but say a lot of how women must preserve, make efforts, and try. In fact, in teaching and discussion sessions with students, for example, in individual social work classes, there are many cases when students discuss unwanted pregnancy and abuse, most of their discussions are also ‘she should do this… do that,’ but not the men… In the way of speaking, it is always the female to be submissive, to find a way to solve the problem while sexual behavior is consensual from both sides. So it is not fair.”—University Lecturer Interview Participant, Central Region, Woman
**Organizational Infrastructure and Resources**	3.4 “My school has the same difficulties, including the lack of experience in hosting big programs or activities. Furthermore, the cooperation between different departments of the school in the process of organizing activities is not at a desirable level, which results in unwanted outcomes. Besides, the school board has not had access to such issues, so I am not sure that the plan for the program will be accepted.” -High School Teacher Focus Group Participant
	3.5 “Actually, my school has a library information center with computers. However, in general, the facilities are not good. I once organized an exam program for students, but the qualities of the network and computers are not stable. Hence, it is quite difficult, based on the actual situation at my school, to implement such online activities." -University Lecturer Focus Group Participant
	3.6 “However, the barrier like I said is that students and teachers have a lot of work and people have to arrange their time. People are interested, but there are many other concerns, so our content maybe not of their priority. Aside from developing and coordinating the program, there are many other things that students do. Well, as you said, they have to work part-time and study for credits. Those things also take up a lot of time. So that's the barrier. Our approach is to put the program in the school based on voluntary and self-disciplined. Hence, it will be a barrier to effective implementation.” -University Lecturer Focus Group Participant
	3.7 “…the problem is that in my university and probably many other universities and schools where I have worked as a communications advisor for teams and groups, the workload is already very heavy. Some teachers find it essential to incorporate it into their lessons, and with or without funding, they still find ways to incorporate it. However, there are many people, including staff and school leaders, who are not that ready if more work does not mean more funding, in which case, it will be a huge barrier. I do not know about Ms. N's school, but in my case, it is very tiring to implement such activities” – High School Teacher Focus Group Participant
**Influential Actors**	3.8 “I see the leaders of my school are very open. The principal and the secretary of the school council are all females. They are very open-minded and understanding. Hence, the reception is not too difficult. Our university is working on communications projects on gender equality, which the Faculty of Journalism is in charge of. From the perspective of the school, when they understand the problem and the project contents, then the reception should not be difficult. Regarding the top-down implementation from the school leaders or asking for the superior's opinion to organize such activities, I think my university is also a very supportive one.” -University Lecturer Focus Group Participant
	3.9 “Implementation through schools’ staff or departments is only suitable for the needs of certain schools if they need educational activities on sexual and reproductive health. However, if you can work with the student union, it will be much more effective. The second challenge, which is also a difficulty for the school, is how to implement this program. The solution is to work with the teachers on sexual and reproductive health education. Teachers can be the ones who proactively educate their students. I think when teachers see the value [of the program], they can implement the program. From my experience, to be sustainable and trendy, the teachers need to integrate the program into the school’s activities. There are many ways to convey the message to the students. From my experience, such an approach is more sustainable and modern." – University Lecturer Focus Group Participant
	3.10 “I see that teachers are the closest people to students, especially headteachers, who are the ones who directly approach, talk, and exchange with students. If a professional person is not available, it is the headteachers who, if trained, can talk to students to catch the signs or detect some cases early. In fact, if there are students who have some signals that are slightly different, the teachers will recognize it, and these teachers know how to talk, which is a great resource.”—CSO Interview Participant, North Region, Man

Several key informants also identified gender norms as an influential element of the institutional culture, reflecting broader normative changes in Vietnam. According to some key informants, male and female students still endorsed more rigid gender norms, characterized by a belief in male privilege and a tendency toward victim blaming (including self-blame) (Table 3, Quote 3.3). By contrast, other informants described gender expectations among students as more equitable, supportive of LGBTQ classmates, and receptive to efforts against gender-based violence.

#### Organizational infrastructure and resources

Key informants identified several influential features of the organizational infrastructure for the implementation of sexual violence prevention programming. A few informants described departments within universities and high schools as siloed, such that limited coordination was a barrier to program implementation (Table 3, Quote 3.4), as was a lack of experience implementing large-scale programming (Table 3, Quote 3.5). Resources also were identified as a salient barrier to sexual violence prevention programming. Key informants noted limited funding and red tape as barriers to implementation, especially in public institutions, and some university lecturers cited technological difficulties, such as inconsistent access to the internet and information/communication technologies, as barriers to students’ participation in online programming (Table 3, Quote 3.5). Informants from high school and university environments discussed limited time and competing priorities among students and teachers as barriers to implementation (Table 3, Quote 3.6–3.7).

#### Influential Actors

In the context of institutional norms and structures, several types of actors were considered important for engaging students in sexual violence prevention and response, and championing program implementation. At universities and high schools, leadership who demonstrated strong buy-in were notable at campuses where successful sexual violence prevention programs were in place (Table 3, Quote 3.8). Within universities, human-resource staff, the Youth Union, school boards or management committees, and student affairs/administrative staff also were identified as facilitating or impeding the institutional response to sexual violence and the implementation of prevention programming. In high schools and universities, several informants cited teachers and other student-facing staff as vital to the implementation process, and a pathway intervention integration and student engagement (Table 3 Quotes 3.9–3.10). These actors operated within the inner setting, autonomously or alongside inner setting leadership, to influence activities and norms, including by championing counter-cultural or deprioritized programs.

### Influential characteristics of individuals

Individuals who interact with the intervention during implementation may exist in the inner and outer settings, and characteristics of those individuals can influence the intervention’s effectiveness (ex., receptivity to change, willingness to engage) and requirements for successful implementation (ex., level of knowledge provided, familiarity with topics). Teachers and lecturers, who are potential facilitators, have varying levels of subject-matter expertise on sexual violence (Table 4, Quote 4.1). Teachers or lecturers with science or social science backgrounds were seen as better prepared to tackle content related to sexual and reproductive health and sexual violence. Also, younger lecturers were considered more open to discussion and perhaps better able to engage with and relate to students’ experiences. Likewise, university students’ area of study was linked with their awareness of sexual violence, willingness to engage in discussions about sexual matters, and involvement with extracurricular activities related to social justice (Table 4, Quote 4.2), as was the urbanicity or rurality of their university. High school and CSO informants often described parents’ more customary attitudes towards sex among young people as a potential barrier to program acceptability (Table 4, Quote 4.3). However, a few CSO informants suggested that parents with more contemporary attitudes could be a potential avenue toward community-level norms change, serving as a bridge to the outer setting (Table 4, Quote 4.4).

**Table 4:** Exemplar Quotes: Influential Characteristics of Individuals

Example
4.1 “Even though the school has cooperated with the regional health clinic to spread awareness about such issues, our teachers lack experience in this. In fact, there is only a school nurse that works with the regional clinic health to organize three training sessions per year, and the teachers are not that interested in this. Even though my school wants to deliver such contents to the students, how to implement them is a big problem for us.” -High School Student Focus Group Participant
4.2 “In my sociology faculty, students are very open when talking to each other or talking to lectures about sex-related issues. This is because our students have had many opportunities to participate in related forums/events and other educational activities, which give them more skills to access official and quality information about sexuality. …However, I think, beside students of my faculty, the rest at the university don’t have much knowledge and understanding about this issue.” -University Lecturer Interview Participant, South Region, Woman
4.3 “A target group that would be surveyed in the program is their parents. I think that is also a barrier because… the community's understanding of this issue is limited. The community has its own customs and habits that are already ingrained in their perception. The general idea of having changes happening overnight, either 100% consensus or enthusiastic participation, also takes time. Especially, it needs the resonance of those who have a voice in the community. If their community is willing to become a leading example, it will be easier to spread and receive the attention of the ethnic minority community.” -University Lecturer Focus Group Participant
4.4 “With the parent's group, when I held a training session on condoms, I recorded a clip and uploaded it on YouTube… A parent called me at and did not quite agree with that at first, but when we talked about it, she thanked me for saying the things that she thought were necessary, but she did not know how to talk about them to her child.” -High School Teacher Focus Group Participant

### Influential characteristics of prevention interventions and implementation processes

Interview and focus group participants, respectively, provided feedback on violence prevention programming generally and GlobalConsent specifically in organizational settings. Participants offered several insights into intervention program structure, often overlapping with views on the process of implementation. Participants’ comments fell into three categories: the intervention medium, intervention delivery, and intervention content. Responses were similar across high schools and university, underlining the needs of students.

#### Intervention medium

Many key informants suggested the medium of the web-based program—online versus in-person—would be effective to raise awareness of sexual violence in universities and high schools, especially through familiar apps and websites (Table 5, Quote 5.1). Key informants noted that virtual programming could fit more easily into students’ schedules and provide greater confidentiality. However, others discussed challenges with virtual programming, including the need for more intentional engagement with students, the difficulty of eliciting feedback from intervention participants, and the time burden outside of school hours (Table 5, Quote 5.2). Responsive to this, some suggested that the program should be implemented in-person, and a few informants felt that one-way delivery of information would be suboptimal, instead suggesting a discussion- or activity-based program. Informants were divided on how to execute sexual violence prevention programming generally, with some suggesting integration into classes, some suggesting a 30-min remote session, and many suggesting more intensive, sustained intervention, including hybrid interventions with in-person and online activities (Table 5, Quotes 5.3–5.5).

**Table 5:** Exemplar Quotes: Influential Characteristics of Prevention Interventions and Implementation Processes Subthemes

Subtheme	Example
**Intervention Medium**	5.1 “I think the most effective way to reach young people is through social media, but it has to be other types of media products that are a bit more interactive." -CSO Interview Participant, Central Region, Woman
	5.2 “Regarding solutions, I could only think of integrating in lessons, since sometimes, even though there are many activities, they still cannot participate due to having to study, hence there should be a subject regarding gender, gender violence, sexual harassment, as well as capacity training sessions or somethings. That would be more specific.” -University Lecturer Interview Participant, South Region, Woman
	5.3 ““My idea is that we can organize activities, for example, by collaborating with school clubs, youth unions, or student unions. I think we should organize competitions. It will increase the effectiveness of the program because young people nowadays hardly have the patience to sit through it all. For example, if they are being asked to sit and watch a whole screening of content, for example, active youth in the Southern region like me, it is very difficult for them to sit through. We can organize contests based on the content from videos that we already had. We can also mobilize the participation of external organizations, for instance, as prize sponsors to raise the award to encourage their participation since I find them nowadays very realistic.”—University Lecturer Focus Group Participant
	5.4 “For my school, I would rate the feasibility to implement at 4. Why so? If it is 100% like what Linh said that it is an extra-curricular activity for them to self-study and self-test, it is possible. In fact, we have done similar activities since 2017. I see students are also quite interested in participating. However, the difficulty here is that it is a 100% online program. Hence, it depends on the students' self-discipline. It will be quite challenging. To my opinion, such extra-curricular activity must be organized by an organization with someone to check and supervise.” -CSO Focus Group Participant
	5.5 “Even each lesson contains short clips, but really, if I have to sit and study continuously, I also feel a bit tired. Additionally, the exercises and I also found the answers to be quite ok, but, if possible, the editor should design more types of exercises. It can be more than just asking and answering questions. It could be games, for example, jigsaw or something else. I enrich the method of playing it a little differently because with what I am participating in, I see it as being not very as diverse as it should be. It needs more variety, that is what I think, to create excitement for students. Otherwise, it will feel a bit boring from beginning to end.” -CSO Focus Group Participant
**Intervention Delivery**	5.6 “If they want to talk, to share, to ask for help, or to let others know to help, for example, or just simply they want to share, they can share with their trusted friends, or even with teachers who they are close with. Because as far as I know, there are lecturers who are trusted by students and can share a lot of things that are not just related to study. In addition, there is also the Student Affairs Department at the university, or (health) medical room also collaborates with the Student Affairs Department for counseling services. They can also share their problems there. Usually, they have the tendency to share with people who are close, or their peers. Counselors, medical staff, or teachers must be extremely close to the students to build trust and safety for the students to share willingly." -CSO Interview Participant, North Region, Woman
	5.7 “During the process of propaganda or information sharing, the communicator needs to be skillful in analyzing for both boys and girls to see their roles. They shouldn’t keep saying ‘Men have to be like this,’ ‘Men have to be like that,’ but invisible, let's make concepts even clearer.” -CSO Interview Participant, North Region, Woman
	5.8 “I also have an additional point, as I mentioned in the chat box, even though I just watched one out of 6 lessons, from my perspective and experience attending online courses, I can see young people like to play games. It is more attractive than a regular method of learning. They can answer multiple-choice questions wrong, but they are allowed to do it again to earn points after each lesson. It is voluntary and more encouraging than forcing by one-time answer only. Hence, they can aim for better final results but are comfortable and fun. If being forced, they may be not interested, or even not attend at all. They may not very like the certificates or similar kinds of recognition. However, if there is some other element that encourages them internally, they will be more patient and proactive.” -University Lecturer Focus Group Participant
	5.9 “…if we run media or social media, we may cooperate with public figures or organizations that are of interest to young people at that time. We can run several media project that propagates sex education content such as talk shows, minigames, etc.… There are certain levels of flexibility for communicating with students of this age, especially in a time when sex education is still something we are aiming at." -CSO Interview Participant, Central Region, Woman
	5.10 “Actually, I see the topics are well linked together. In my opinion, the topics are quite close. However, there is one small thing. I do not know how everyone learned all 3 lessons. I think it's long. Even each lesson contains short clips, but really, if I have to sit and study continuously, I also feel a bit tired. Additionally, the exercises and I also found the answers to be quite ok, but, if possible, the editor should design more types of exercises. It can be more than just asking and answering questions. It could be games, for example, jigsaw or something else. I enrich the method of playing it a little differently because with what I am participating in, I see it as being not very as diverse as it should be. It needs more variety, that is what I think, to create excitement for students. Otherwise, it will feel a bit boring from beginning to end.” -University Lecturer Focus Group Participant
	5.11 “We must mobilize adolescents in the provision of information and change their behaviors toward a positive direction for both males and females. If they are [seen as] the agents of change, it will work. However, if we still educate in a dogmatic form and still from the perspective of non-insiders, [it will be ineffective]. We are not insiders. It is more effective for teenagers to talk about the issue themselves." – CSO Interview Participant, North Region, Woman
	5.12 “I also agree with the previous opinion, that if we just leave it to student’s self-discipline, it is also very challenging, as I suggested in my comments on the videos. I just want to remind you that the schools also have a lot of work. Hence, such a program will be more feasible to implement if the initiative can be taken to the school's clubs or organization, for example, youth union or student’s life with proper organization and management or being integrated into life skills programs. If you just introduce and let people join the program, I think it is not effective." – University Lecturer Focus Group Participant
**Intervention Content**	5.13 “Actually, I have participated in the program which I find attractive, especially the teenager-friendly language, and natural acts in theatrical play like a normal coffee chat. That's what I really like. It is very suitable for young people and everyone can get the general idea of such a clip. That is the first thing. The second thing is exercise. There is a rule for answers. I think it's very good. I read their answers whose arguments were based on evidence… Another good thing is that the contents’ length is usually 30 s to 1 min instead of 3–5 min. However, if I want to listen faster to save me some time, I may want to adjust the speed of the videos just like Youtube’s function. That is about me only. So, those are some of my opinions. In general, I find the program very interesting. This is the first well-organized program that I have seen.”—University Lecturer Focus Group Participant
	5.14 “The university environment is very open. However, earning students' trust so they can share their stories, is a whole process along with a lot of activities. It should not be separate activities, i.e., training, but in cooperation with communications or events. Such events with communication and counseling integrated should be further promoted.” -CSO Interview Participants, North Region, Woman
	5.15 “I find this knowledge very useful for all of the students. However, it still only focuses on relationships among male vs female students but neglects external elements in emotional and sexual relationships. We still only consider the relationships among students but there are also other parties in the university, for example, male lecturers or older men.”—University Lecturer Focus Group Participant
	5.16 “There are pieces of information included in these six videos that are more suitable for university students than high schoolers. And children with a 4-year age gap have quite different perspectives on life and sex; hence I suggest that we modify some of the content to make it more accessible to the age group.” – High School Teacher Focus Group Participant
	5.17 “Within the group of men, the intervention is not just male intervention since I think the intervention between men and men is very one-sided. And for interventions that include a male target group, there should be also side groups such as women or girls, so that they can learn together, share and discuss with each other. It will be easier for them to listen to each other as well as to understand the needs or expectations of other groups and gender For example, among men vs men, they may assume women’s expectations which will be less convincing than an intervention designed that includes both men and women. Because there is nothing more convincing than the females themselves sharing their expectations with the group of men about how to reduce sexual violence or violent behavior in general, or what they expect in their partners, lovers, or future husbands. Such intervention is more practical than ones that only men vs men. I find them quite separate with no interaction and missing open views from two sides.” -CSO Interview Participant, North Region, Woman

#### Intervention delivery

Delivery of the intervention—the means through which information is delivered, such as the facilitator, didactic versus interactive, and means to engage students—was underscored as requiring trusted sources (Table 5, Quote 5.6). Intervention facilitators were cited as needing training on how to communicate concepts related to violence (Table 5, Quote 5.7). Given the digital format, several participants noted the potential to deliver intervention materials through numerous mechanisms and the overall flexibility of a web-based platform, and conveyed that integrating other types of activities, such as games and talk-show-style content, may be beneficial (Table 5, Quote 5.8–5.9). These approaches were recommended to offset the abundant information presented throughout the program (Table 5, Quote 5.10). To facilitate students’ engagement in the content, participants underscored the importance of peer education (Table 5, Quote 5.11). Participants also expected difficulty engaging students without incentives or integration into school activities (Table 5, Quote 5.12).

#### Intervention content

Participants generally liked the content of the GlobalConsent program (Table 5, Quote 5.13). Participants underscored the need to provide information on ancillary support to students who engage in the intervention, such as sexual violence support services (Table 5, Quote 5.14). Other recommended new content on relationships that are not only student–student, but involve teachers (Table 5, Quote 5.15). Some content was noted as too advanced for high school students, and required adaptation to that audience (Table 5, Quote 5.16). Several participants also encouraged adding content for women in the intervention (Table 5, Quote 5.17).

## Discussion

### Summary and interpretation

This qualitative study is the first to explore facilitators and barriers to sexual violence prevention program implementation, according to key informants representing three youth-centered organizational settings across Vietnam. This study is timely, given the high prevalence of sexual violence among young people in Vietnam [5] and recent evidence for the efficacy of GlobalConsent among university men in Hanoi [9, 21], suggesting a need to bridge the gap between program efficacy and real-world effectiveness [24, 25]. Our combined deductive/inductive analytical strategy allowed us to apply the Consolidated Framework for Implementation Research [51] to understand the context of sexual violence prevention in Vietnam. This qualitative study is the first to explore facilitators and barriers to sexual violence prevention program implementation, according to key informants representing three youth-focused centered organizational settings across Vietnam. Given the focus of this study on considerations for the implementation (at scale) of adapted, efficacious sexual-violence prevention interventions [21], this study extends prior research on the adaptation of sexual-violence prevention interventions to LMIC settings [56, 57]. This study also is timely, given the high prevalence of sexual violence among young people in Vietnam [5] and recent evidence for the efficacy of GlobalConsent among university men in Hanoi [9, 21], suggesting a need to bridge the gap between program efficacy and real-world effectiveness [24, 25]. Our combined deductive/inductive analytical strategy allowed us to apply the Consolidated Framework for Implementation Research [51, 52, 58] to understand the context of sexual violence prevention in Vietnam.

Key informant interviews and group discussions allowed the team to explore deductively the salience of major CFIR domains while identifying inductively salient sub-themes within CFIR domains. In the outer setting, one commonly perceived barrier to implementation across universities, high schools, and CSOs was the co-existence of more open sexual norms among young people alongside traditional gender norms favoring men’s sexual privilege. This finding corroborates qualitative research among university men in Hanoi and suggests that more rapidly changing sexual norms than gender norms may be normalizing non-consent and sexual coercion among young people [11, 13]. A second common outer-setting influence was ostensibly ambiguous and poorly implemented laws on sexual violence. This perception does not align with recent efforts to reform national laws and policies on sexual violence but does suggest the need for prevention programming that raises awareness of legal definitions of sexual violence and that promotes empathy for victims [9]. Other salient outer-setting influences were government ministries, which were seen simultaneously as bureaucratic and potential allies, external subject-matter experts, and the media. Key informants’ perceptions of the mixed-influence of the media corroborates findings among high-school and university men in Vietnam that the internet is a source of general content about sex, and of violent sexually explicit material that predicts sexual violence against women [59, 60].

Inner-setting influences included institutional cultures that varied in their openness to discuss sexual violence and in their prevailing gender norms, extent of departmental coordination for institution-wide programming, limited funding and ‘red tape’ especially in public institutions, inconsistent access to resources, and competing demands on time among students and teachers. Implementation research of sexual violence programs in the United States has underscored the importance of ‘fit’ between the program and the adopting organization’s values [61]; however, implementation with fidelity was reported to be possible with modest investments in training and technical assistance. The limited funding of LMIC institutional settings may require more start-up investment and training for implementation with fidelity.

In these organizational contexts, several actors were considered influential, including supportive institutional leaders, human-resource staff, the Youth Union, and student-facing staff. Important characteristics of individuals for implementation included subject-matter expertise, science or social science training, younger age, and more contemporary attitudes about sex. Such findings underscore the potential differences in attitudes between older and younger staff and the widespread need for implementation training specific to sexual violence prevention. Regarding characteristics of sexual violence prevention programming, some participants preferred online formats for busy students while others suggested hybrid or in-person formats, peer education, and incentives. Participants generally accepted the content of GlobalConsent and suggested adding more content for women, ancillary support services, and adapted content for high-school students. Key informants’ favorable views of GlobalConsent’s content corroborate high satisfaction ratings from university men (7.8 on a 1–10 scale).

### Limitations and Strengths

The present study faced some challenges that required strategic solutions. Stakeholder recruitment was more difficult in Central and South Vietnam, so the team diversified its networks for snowball sampling and recruitment. CCIHP, for example, approached staff in youth-engaged CSOs who were not sexual violence experts and asked their help to expand the network for recruitment. The key informant interview guide did not follow the CFIR framework; whereas, the discussion guide did. This lack of comparability in the guides actually allowed the team to use inductive and deductive strategies to understand the implementation context and to triangulate data from different approaches to ensure the validity of both sources. The CFIR domains were employed to contextualize findings to intervention implementation, but not all constructs in each CFIR domain were employed; this flexibility enabled the team to identify salient emergent constructs among participants. The lack of some CFIR sub-domain constructs may impact comparability of this work to other implementation efforts. Finally, the data on intervention characteristics and implementation process were thinner, in part as a result of the organization of the key informant interview guide. Therefore, interpretations of the salience of these domains should be made with caution. Otherwise, rich data were available on other major domains of the CFIR.

### Implications for implementation/effectiveness research and scaling efficacious sexual violence prevention interventions

Findings from this study offer several insights about promising strategies to test in implementation/ effectiveness research on sexual violence prevention programming in Vietnam. First, implementation researchers should be sensitive to prevailing gender norms that privilege men as an important element of the outer setting, which may influence organizational decisions about program uptake and about implementation strategies to increase program acceptability. Second, engaging external subject-matter experts could address low legal knowledge about sexual violence, misperceptions of low prevalence, and reputational concerns among institutional leaders. Third, subject-matter experts may be useful to train internal champions in program implementation as well as to provide on-going technical support. Protecting the time of trained staff could help to allocate human resources needed for implementation fidelity, to promote open discussion of sexual violence among student-facing staff, and to engage students in program activities. Fourth, strategies to address the competing demands on students’ time may be needed for program uptake, retention, and completion. Possible strategies may include a school-based orientation to the program, regular participation reminders, and incentives to complete the program. Testing these implementation strategies as a ‘bundle’ may help to address the multilevel barriers to implementation that key informants identified in the present study, and reduce the efficacy-effectiveness gap of promising programs, like GlobalConsent. Finally, the incorporation of cost-effectiveness research when evaluating gender-based violence interventions is overlooked and greatly needed to assess the practical feasibility of various intervention approaches in real-world environments. Based on the findings from the present study, testing the incremental cost effectiveness of more intensive, ‘bundled’ implementation strategies versus less intensive, ‘standard’ implementation strategies also would provide practical guidance about the resources needed for sustained implementation.

## Conclusion

Implementation of sexual violence prevention programs in youth-focused organizations in Vietnam requires multilevel strategies that connect outer-setting organizational allies and subject-matter experts with supportive inner-setting leaders and student-facing champions to overcome normative and organizational constraints, and thereby, to deliver institution-wide prevention program with sustainment. Understanding the costs of implementing at scale efficacious sexual violence prevention interventions also is needed for uptake and sustained implementation in LMIC settings.

## Supplementary Information

**Additional file 1.**

## Data Availability

The datasets used and/or analyzed during the current study are available from the corresponding author on reasonable request**.**

## Abbreviations

MOLISA: Ministry of Labor, Invalids, and Social Affairs
LMICs: Low- and Middle-Income Countries
EBIs: Evidence-Based Interventions
CFIR: Consolidated Framework for Implementation Research
GSO: General Statistical Office
UNDP: United Nations Development Program
CSO: Civil Society Organization
FGD: Focus Group Discussion
INGO: International Non-Governmental Organization

## Declarations

### Authors’ Information

Kathryn Yount, PhD, is Asa Griggs Candler Professor of Global Health (2012) and Professor of Global Health and Sociology (2015) at Emory University. Her research centers on the social determinants of women’s health, including mixed-methods evaluations of social-norms and empowerment-based programs to reduce gender-based violence and health disparities in underserved populations. She has been funded continuously since 2002 from U.S. federal agencies, private foundations, and foreign agencies to work in parts of Asia, Latin America, the Middle East, Sub-Saharan Africa, and underserved communities in Atlanta. These collaborations have culminated in more than 250 publications in the social sciences and global health. She is recipient of the Women of Emory Award for Mentoring (2016), the Marion V. Creekmore Award for Internationalization (2022), and the Eleanor Main Graduate Mentor Award (2023).

Katherine M. Anderson, MPH, is a Doctoral Student in the Department of Behavioral, Social, and Health Education Sciences at the Emory University Rollins School of Public. Ms. Anderson has significant experience engaging in research on HIV treatment and prevention, access to health care services, health care interactions, trauma-informed care, populations experiencing violence, and violence prevention, both domestically and internationally.

Irina Bergenfeld, MPH, is a violence prevention researcher earning a PhD in Global Health and Development at Emory University, where she received the Woodruff and Paul J. Coverdell Fellowships. Her work focuses on gender-based violence and women's empowerment, including girls' education, child and forced marriage, sexual violence, and intimate partner violence. As a former Program Associate at Emory, she coordinated and supported research teams across the US, Jordan, Bangladesh, Vietnam, Kenya, and Nepal. Her research focuses on the measurement of gender-based violence and its correlates.

Quach Thu Trang, MA, has 22-years working experience in qualitative research and program management. Her prior experience in the field of gender-based violence includes formative studies and the local production of GlobalConsent – a web-based training program on sexual violence prevention for male university students (Emory University/CCIHP, 2017–2021); a gender-based violence integrated manual development and training delivery for rehabilitation health staff (USAID/CCIHP/I-Thrive 2019–2021); Southeast Asia Regional Curriculum Development Team member on masculinities and gender-based violence (UNDP/P4P/2010 – 2012); and Development Group Member for WHO Guideline on Sexual and Reproductive health and Rights of women living with HIV (WHO Geneva, 2016).
