# Supplementary material for: Preventing sexual violence in Vietnam: qualitative findings from high school, university, and civil society key informants across regions
Source: BMC Public Health. 2023 Jun 10;23:1114. doi: 10.1186/s12889-023-15973-5 (PMC10256971; doi:10.1186/s12889-023-15973-5)
Supplement: Supplementary file 1 — Additional file 1. [file 12889_2023_15973_MOESM1_ESM.zip › SEANET_FG GuideR3.docx]

**Southeast Asia Violence Prevention Network (SEANET) Project – Vietnam Stakeholders**

**Focus Group Discussion Guide**

**Feedback on GlobalConsent Implementation Considerations**

**Group ID #: [___________________] Date: ___________________­_________**

**Moderator Name: _____________________ FGD location: ______________________**

**Start time: :**

**(hour) (minutes)**

**End time: :**

**(hour) (minutes)**

**Audio file #: [___________________]**

**Notes upon completion:**

**INTRODUCTION AND WARM-UP**

*[Facilitators greet participants and introduce themselves.]*

Hi, my name is [NAME] from the Center for Creative Initiatives in Health and Population, or CCIHP. We are conducting focus groups with educators and members of youth-focused non-governmental organizations to gain a better understanding of your views about the GlobalConsent program you have viewed and its feasibility and acceptability for implementation in your organization. I shared a consent form with you already, but before we begin, let me describe the study now and confirm your consent to participate.

*[Facilitator: Please complete the verbal consent process and digitally record verbal informed consent.]*

If you agree to take part, the information you provide will be used for research purposes. We ask for your support by responding to the questions as honestly and fully as possible. To respect the confidentiality of the other participants, we ask you to please not share with others what is said during this discussion. This group discussion is not an assessment of your efforts, and there are no right or wrong answers. No names will be linked to any responses or data. You may withdraw from the study at any time, and if there are questions that you would prefer not to answer, we respect your right not to answer them. There is no foreseen cost to your participation in this study, except for the 45 to 60 minutes you will spend with our study team. If you have questions about the research in general, about your role in the study, or about your rights as a participant in this study, please feel free to contact Dr. Tran Hung Minh at (84-4) 35770261.

Do you have any questions about this interview/research?

*[Facilitator please answer any questions. When all questions are answered, please have all participants introduce themselves and indicate whether they consent to participate and record.]*

Are you willing to participate?

*[Document in table below and thank participants who refuse for their time.]*

Are you willing to have our group discussion recorded?

*[Document in table below and thank participants who refuse for their time.]*

| **Participant** | **Organization** | **Sector**  **(uni, high-school, CSO)** | **Role** | **Age, years** | **Gender (man, woman, non-binary)** | **Consent to participate** | **Consent to record** |
| --- | --- | --- | --- | --- | --- | --- | --- |
| **1** |  |  |  |  |  |  |  |
| **2** |  |  |  |  |  |  |  |
| **3** |  |  |  |  |  |  |  |
| **4** |  |  |  |  |  |  |  |
| **5** |  |  |  |  |  |  |  |
| **6** |  |  |  |  |  |  |  |
| **7** |  |  |  |  |  |  |  |
| **8** |  |  |  |  |  |  |  |
| **9** |  |  |  |  |  |  |  |
| **10** |  |  |  |  |  |  |  |

*For participants who agree, Facilitator states: “Thank you for agreeing to participate. I appreciate your help. Is everyone ready to begin? Great, let’s begin.”*

*First, we want everyone to feel comfortable. Again, there are no right or wrong answers here, we just want to hear your opinions about the program we have presented to you. Therefore, please be respectful of the other participants’ thoughts and opinions. We can disagree as long as we do so in a constructive way. Second, we want to hear from each one of you, so if you notice that you have been contributing a lot to the discussion, please take a step back and let us hear from someone who may have been speaking less. Third, you may feel free to leave the session if you need to use the restroom or take a short break. Finally, your participation in this focus group discussion is voluntary, so if the content makes you feel uncomfortable or distressed, you are free to take a break from the discussion or to withdraw from the study. Please mute your microphone when you are not speaking and keep your video on unless you do not wish to be recorded. Does anybody have any other ground rules they would like to add to ensure that this discussion is respectful and productive?*

*[Facilitator, please see if any participants have ground rules to add.]*

**Group Discussion about Individual Work**

*[Each participant will have received the questions below in advance and will have had an opportunity to complete an implementation matrix for their own organization. The focus group facilitator now goes back to each question and invites participants to talk about their individual responses. The goal will be to identify institutional characteristics (norms, policies, key stakeholders, current curriculum, etc) that are barriers and/or facilitators to implementing GlobalConsent within specific institutions and some or all institutions. The group will work together to fill out a joint implementation matrix with institution-specific and common contextual facilitators and barriers]*

*Let’s go around and discuss your responses to question #1. I will record them here in a single document so we can easily see the similarities and differences.*

*1. As you have seen, GlobalConsent is a web-based program that can be delivered to smartphones, other mobile devices, or computers. Now, please think about the environment at your institution or organization. On a scale from 1 to 5, with 1 meaning not at all feasible and 5 meaning extremely feasible), how FEASIBLE would it be to deliver GlobalConsent to [all students at your institution/all youth members of your organization]? Please think about how you would identify (find), reach (deliver), and retain all of the recipients of the GlobalConsent program to completion. Please clarify all of the reasons for your answer. Probe until no one in the group provides any other reasons.*

*2. Now, please think about the different stakeholder groups inside your institution, such as students, parents, educators, administrative leaders, or others. On a scale from 1 meaning not at all acceptable to 5 meaning extremely acceptable, how ACCEPTABLE would the GlobalConsent program be to each of these different stakeholder groups inside your institution? Please clarify the reasons for your answers.*

*Students:*

*Parents:*

*Educators:*

*Administrative leaders:*

*Others [___________]:*

*3. Now, please think about the different stakeholder groups outside your institution. On a scale from 1 meaning not at all acceptable to 5 meaning extremely acceptable, how ACCEPTABLE would the GlobalConsent program be to each of these different stakeholder groups outside your institution? Please clarify the reasons for your answers.*

*Group 1 [_______]:*

*Group 2 [_______]:*

*Group 3 [_______]:*

*Group 4 [_______]:*

*Group 5 [_______]:*

*4. Now, please describe all of the characteristics of your institution that would ENABLE GlobalConsent to be implemented at your institution?*

*5. What are the characteristics of your institution that would create BARRIERS to implement GlobalConsent?*

**Questions to the Group**

*6. Now, please describe all of the characteristics of the environment outside your institution that would ENABLE GlobalConsent to be implemented at your institution? Probe until no more characteristics are provided. Probe: Focusing on influences that would help implement GlobalConsent, what organizations outside of your institution influence activities like GlobalConsent at your institution? Probe: What rules or regulations does your institution have to follow that might influence implementing GlobalConsent? Probe: How supportive do you think the community outside of your institution is of implementing something like GlobalConsent?*

*7. Ok, now, what are all of the characteristics of the environment outside your institution that would create BARRIERS to implement GlobalConsent? Probe until no more characteristics are provided. Probe: Focusing on influences that would NOT help implement GlobalConsent, What organizations outside of your institution influence activities like GlobalConsent at your institution? Probe: What rules or regulations does your institution have to follow that might influence implementing GlobalConsent? Probe: How supportive do you think the community outside of your institution is of implementing something like GlobalConsent?*

*8. Thank you, now please tell me which modules, if any, may be particularly SENSITIVE at your institution? Probe: What other modules, if any? Probe: For whom would module [#] about [topic] be sensitive? Probe: For what reasons? Probe: What could be done to make this module or segment less sensitive? Probe until no more modules or segments are mentioned.*

*9. Now, please tell me which modules, if any would be particularly WELCOMED? What other modules, if any? Probe: For whom would module [#] about [topic] be welcomed? Probe: For what reasons? Probe until no more modules or segments are mentioned and no more reasons are given.*

*10. What other characteristics of the of the GlobalConsent intervention would be helpful in implementing GlobalConsent at your institution? Probe: What characteristics of the intervention would make it difficult to implement GlobalConsent at your institution? Probe: What about GlobalConsent could be changed to address these difficulties?*

[*Facilitator says,* “*That was my last question. If you have any questions later, feel free to contact me. We appreciate the information you have provided and your willingness to share so openly with us and the rest of the group. Please remember the agreement you made not to discuss the information shared today anywhere, or with anyone outside of this room. At this time, we’d like to ask you to re-affirm your commitment to maintaining this confidentiality by raising your hand. Thanks again for participating in the discussion today! We appreciate you sharing your thoughts and guidance.”]*

**[END RECORDING]**
