# Supplementary material for: Preventing sexual violence in Vietnam: qualitative findings from high school, university, and civil society key informants across regions
Source: BMC Public Health. 2023 Jun 10;23:1114. doi: 10.1186/s12889-023-15973-5 (PMC10256971; doi:10.1186/s12889-023-15973-5)
Supplement: Supplementary file 1 — Additional file 1. [file 12889_2023_15973_MOESM1_ESM.zip › SEANET_KII GuideR3.docx]

**SEXUAL VIOLENCE PREVENTION LEARNING COLLABORATIVE**

**GUIDE FOR KEY INFORMANT INTERVIEWS**

**Participant ID #: [________________] Date: _________________________________**

**Interviewer Name: ________________________ Interview location: ______________________**

**Start time: :**

**(hour) (minutes)**

**End time: :**

**(hour) (minutes)**

**Audio file #: [___________________]**

**Notes upon completion:**

**INTRODUCTION**

Hi, my name is [name] from CCIHP. We are conducting interviews with educators and members of youth-focused non-governmental organizations to gain a better understanding of sexual violence among young people and what we can be done to prevent it. I shared a consent form with you already, but before we begin, let me describe the study now and clarify your consent to participate.

**INTERVIEWER: Please complete the verbal consent process and digitally record verbal informed consent.**

If you agree to participate, the information you provide will be used for research purposes. We ask for your support by responding to the questions as honestly and fully as possible. Your responses will be kept COMPLETELY CONFIDENTIAL. This is not an assessment of your efforts and there are no right or wrong answers. There will be no names that will be linked to any responses or data. You may withdraw from the study at any time and if there are questions that you would prefer not to answer then we respect your right not to answer them. There is no foreseen cost to your participation, except for the 45 to 60 minutes you will spend with our study team. We may also contact you again to ask you to join a 60 to 90 minute focus group discussion with other participants. If you have questions about the research in general, about your role in the study, or about your rights as a participant in this study, please feel free to contact Dr. Tran Hung Minh at (84-4) 35770261. Do you have any questions about this interview/research? Are you willing to participate?

**Check for participant consent ____**

**PARTICIPANT DEMOGRAPHIC QUESTIONS**

- What school/organization do you work with?
- (For NGOs) What is the objective of this organization?
- With what ages does your organization/school mainly work?
- How many years have you worked there?
- What is your role in this organization?
- How many years have you been in this role?
- How many years have you worked in this sector (education, activism, etc.)?

**GUIDED QUESTION SETS**

**Perceptions about the nature and scope of sexual violence among young people**

**READ ALOUD**: First, we would like to understand your perceptions of what happens among young people and of social expectations of young people in your organization.

1. How common are sexual relationships among young people at your school/organization? How have relationships among young people changed over the past few decades?
2. What are the social expectations placed on young women regarding sex and sexual relationships among young people? (Probe: How do young women respond to these expectations?)
3. What are the social expectations of young men, for example among adults or young men’s peers, about young men’s sexual behavior and sexual relationships with women?

**Probe**: How do young men respond to these expectations?)

1. How do you define sexual consent?

**Probes**: Under what circumstances is consent possible/not possible? How do you think most people (youth, adults) define sexual consent?)

1. How do you define sexual violence?

**Probes**: What kinds of acts constitute sexual violence? How do you think other people define sexual violence? How common is sexual violence among young people in your organization? What specific types of sexual violence commonly occur among young people in your organization?

1. How do you define sexual coercion?

**Probes**: What kinds of acts constitute sexual coercion? How do you think other people define sexual coercion? How common is sexual coercion among young people in your organization? What specific types of sexual coercion commonly occur among youth in your organization?

**Perceived reasons for sexual violence perpetration**

1. As far as you know, what is the most typical situation when sexual violence happens among youth?

**Probe**: What other situations can lead to sexual violence?

1. In your opinion, what usually is the main cause of sexual violence? Who most often is involved? Who typically is to blame?

**Probe**: Would most people agree with you? Why or why not?

1. What are the outcomes for the perpetrator? What are the outcomes for the victim?
2. What are the most common beliefs and misconceptions about sexual violence?
3. How does sexual violence among youth differ from sexual violence among adults, if at all?

**Strategies to prevent sexual violence and sexual coercion**

1. What are ways that youth can learn more about healthy dating and relationships?
2. What do youth who have had bad experiences in dating relationships do if they want to talk about it?
3. What resources are needed to address sexual violence among youth?
4. What role do men and boys play in preventing sexual violence?
5. What role does the educational/NGO sector play in preventing sexual violence among youth?
6. What else would you like to add that we have not yet discussed today?

**Potential barriers to sexual violence prevention at [school/organization]**

1. What opportunities do you see to address sexual violence at your institution?
2. What are the major barriers to address sexual violence at your institution?

**Probe**: Given your experience, how can these barriers best be overcome?

Thank you for speaking with me today. Your answers have been very helpful*.*

[END RECORDING]
