# Supplementary material for: Preventing sexual violence in Vietnam: qualitative findings from high school, university, and civil society key informants across regions
Source: BMC Public Health. 2023 Jun 10;23:1114. doi: 10.1186/s12889-023-15973-5 (PMC10256971; doi:10.1186/s12889-023-15973-5)
Supplement: Supplementary file 1 — Additional file 1. [file 12889_2023_15973_MOESM1_ESM.zip › SEANET_Reading GuideR3.docx]

**Southeast Asia Violence Prevention Network (SEANET) Project– Vietnam Stakeholders**

**Individual Focus-Group Participant Form**

**Feedback on GlobalConsent—Implementation Considerations**

**Individual ID #: [________________] Date: ________________________**

**(To be completed by CCIHP) (To be completed by participant)**

**INTRODUCTION**

Thank you for agreeing to take part in our study. We will be conducting focus groups with educators and members of youth-focused non-governmental organizations to understand your views on the Global-Consent program that you are about to view and its feasibility and acceptability for implementation in your organization. As you view the GlobalConsent program, we ask that you fill out the following four questions in advance of our discussion on [INSERT DATE], 2022. There are no right or wrong answers; we are interested in your opinion about how GlobalConsent can be implemented at institutions like yours.

**MODULE-SPECIFIC QUESTION**

Now, after you view each module, please respond briefly to the question below. Please write your answer in the space provided.

| **Module** | **Question: If you were invited to implement this module to young people at your institution, what considerations, whether positive or negative, would you have? (*You could write down either the positive or the negative impression or both of each module*)** | |
| --- | --- | --- |
|  | Positive impression/consideration | Negative impression/ consideration |
| **1 – sexual consent** |  |  |
| **2 – myths on rape and gender role** |  |  |
| **3 – communication barriers...** |  |  |
| **4 – alcohol influence...** |  |  |
| **5 – empathy with victims...** |  |  |
| **6 – observersreactions...** |  |  |

Thank you for viewing the GlobalConsent program. Now that you have finished viewing it, please respond to the following questions about the program as a whole. Please write your response below to each question.

**OVERALL QUESTSIONS ABOUT GLOBALCONSENT**

1. As you have seen, GlobalConsent is a web-based program that can be delivered to smartphones, other mobile devices, or computers. Now, please think about the environment at your institution. On a scale from 1 to 5, with 1 meaning not at all feasible and 5 meaning extremely feasible, how FEASIBLE would it be to deliver GlobalConsent to all young men at your institution? Please think about how you would identify (or find), reach (deliver the program), and retain all of the recipients of the GlobalConsent program to completion. Please clarify all of the reasons for your answer.

**1 2 3 4 5**

**Not at all Moderately Extremely**

**Feasible Feasible Feasible**

**Reasons for Response:**

2. Now, please think about the different stakeholder groups **inside your institution**, such as students, parents, educators, administrative leaders, or others. On a scale from 1 meaning “not at all acceptable” to 5 meaning “extremely acceptable,” how **ACCEPTABLE** would the GlobalConsent program be to each of these different stakeholder groups at your institution? In the space below, please clarify the reasons for your answers.

**1 2 3 4 5**

**Not at all Moderately Extremely**

**Acceptable** **Acceptable** **Acceptable**

| Stakeholder group inside institution | 1 | 2 | 3 | 4 | 5 | Reasons for response |
| --- | --- | --- | --- | --- | --- | --- |
| Students |  |  |  |  |  |  |
| Parents |  |  |  |  |  |  |
| Educators |  |  |  |  |  |  |
| Administrative leaders |  |  |  |  |  |  |
| Others (if any) |  |  |  |  |  |  |

3. Now, please think about the different stakeholder groups **outside your institution**. On a scale from 1 meaning “not at all acceptable” to 5 meaning “extremely acceptable,” how **ACCEPTABLE** would the GlobalConsent program be to each of these different stakeholder groups at your institution? In the space below, please clarify the reasons for your answers.

**1 2 3 4 5**

**Not at all Moderately Extremely**

**Acceptable** **Acceptable** **Acceptable**

| Stakeholder group outside institution | 1 | 2 | 3 | 4 | 5 | Reasons for response |
| --- | --- | --- | --- | --- | --- | --- |
| Group 1: Government administration agency (e.g. MOET) |  |  |  |  |  |  |
| Group 2: Media |  |  |  |  |  |  |
| Group 3: People in communities/ society in general |  |  |  |  |  |  |
| Group 4: |  |  |  |  |  |  |
| Others (if any) |  |  |  |  |  |  |

4. Now, please describe all of the characteristics of your institution that would **ENABLE** GlobalConsent to be implemented at your institution? Probe until no more characteristics are provided.

**Response:**

5. What are the characteristics of your institution that would create **BARRIERS** to implement GlobalConsent? Probe until no more characteristics are provided.

**Response:**

**Thank you for viewing the GlobalConsent program and taking the time to respond to our questions. We look forward to your participation in the focus group discussion.**
